# Hospital discharge codes and substantial underreporting of congenital heart disease

**DOI:** 10.1016/j.ijcchd.2022.100320

**Published:** 2022-01-04

**Authors:** Jason Chami, Calum Nicholson, Geoff Strange, David Baker, Rachael Cordina, David S. Celermajer

**Affiliations:** a Sydney Medical School, University of Sydney, Camperdown, NSW, 2006, Australia; b Heart Research Institute, 7 Eliza St, Newtown, NSW, 2042, Australia; c School of Medicine, University of Notre Dame Australia, 21 Henry St, Fremantle, WA, 6160, Australia; d Royal Prince Alfred Hospital, Missenden Rd, Camperdown, NSW, 2050, Australia

**Keywords:** Adult congenital heart disease, Congenital heart disease, Quality of care

## Abstract

**Background:**

Hospital discharge codes are relied upon for research, accounting/invoicing and health systems planning. Congenital heart disease (CHD), however, is uniquely difficult for non-cardiologists to code due to the rarity, variety and complexity of lesions. It is therefore important that the accuracy of hospital discharge codes is regularly checked to ensure that the prevalence and burden of CHD is being correctly estimated and recorded.

**Methods and results:**

We identified all inpatient admissions of adults with CHD to Royal Prince Alfred Hospital in Sydney, Australia from January 2018 to March 2021 (257 admissions, 106 unique patients). The associated discharge coding summaries were extracted and compared to the codes in the separately collected and audited Adult CHD database. Only a quarter of discharge coding summaries contained any diagnosis of CHD, and just one-tenth accurately recorded all appropriate CHD diagnoses. Patients with simple lesions were most likely to have a coded diagnosis of CHD, while those with moderate and complex lesions were much less likely. Moreover, patients admitted under a cardiovascular specialty were twice as likely to have a coded diagnosis of CHD, compared with those admitted under non-cardiovascular specialties (p ​= ​0.006). Overall, less than half of patients had any hospital-coded diagnosis of CHD in any admission over the three-year study period.

**Conclusions:**

Hospital discharge coding dramatically underreports CHD, especially for patients with moderate and severe CHD lesions and for admissions under non-cardiovascular specialties. This suggests that discharge coding-based estimates of the burden of CHD on hospitals and health systems may be substantially underestimated.

## Highlights

• We extracted 257 hospital admissions with congenital heart disease (CHD).
• Only a quarter of discharge summaries contained any diagnosis of CHD.
• Only one-tenth contained all appropriate CHD diagnoses.
• Patients with simple lesions were coded best; moderate and complex lesions worst.
• Admissions under Cardiology teams were twice as likely to have a coded CHD diagnosis.

## 1 Introduction

As electronic medical records have grown in size, complexity and importance, so too have the medical coding systems that power them. Today, almost every developed health care system discharges patients along with a set of codes which describe the patient’s diagnoses and comorbidities, as well as any procedures or surgeries performed during their stay. These discharge coding summaries are used for research into prevalence and outcomes of diseases, by hospital administrators for planning and occasionally for invoicing, and by health system planners to allocate funding and human resources. Discharge coding in Australia is performed by specially trained staff, using a modification of the International Classification of Diseases (ICD) known locally as ICD-10-AM [1]. Coding staff are required to have completed tertiary health sciences education and comprehensive training in ICD-10-AM medical terminology, including specific training for cardiovascular disease. Discharge coding summaries are therefore assumed to be both comprehensive and accurate.

Congenital heart disease (CHD), however, poses a unique challenge to discharge coders [2]. Not only are the lesions complex, with small anatomical differences resulting in different physiology and treatment protocols, but they are also numerous and frequently rare, making coding particularly difficult for non-cardiologists. Moreover, while ICD-10-AM was sufficient for administrative purposes in the early 2000s, hospital databases are increasingly relied upon for epidemiological research, where a great deal more detail is required. Advances in imaging since then have also revealed much more detail that cannot necessarily be captured by relatively coarse diagnostic codes. Finally, with improved treatment, more children with CHD are now able to live until adulthood, where their progress and care must be tracked and optimized over the long term [3]. Such is the step-change in understanding, and the increase in case numbers, that over the last 20 years several entirely new coding systems have been devised specifically for congenital heart disease [[4], [5], [6], [7]].

We were prompted to examine the accuracy of CHD discharge codes by a government review of health services planning, which relied on coding accuracy to understand the numbers and distribution of CHDs in New South Wales (NSW), the most populous state in Australia. Our hospital has the largest adult CHD quaternary referral service in NSW. We hypothesized that CHDs might be underreported and/or inaccurately reported in our hospital discharge coding summaries. This would have potentially serious implications for research, investment and health policy.

## 2 Methods

### 2.1 Data collection

**Fig. 1:**
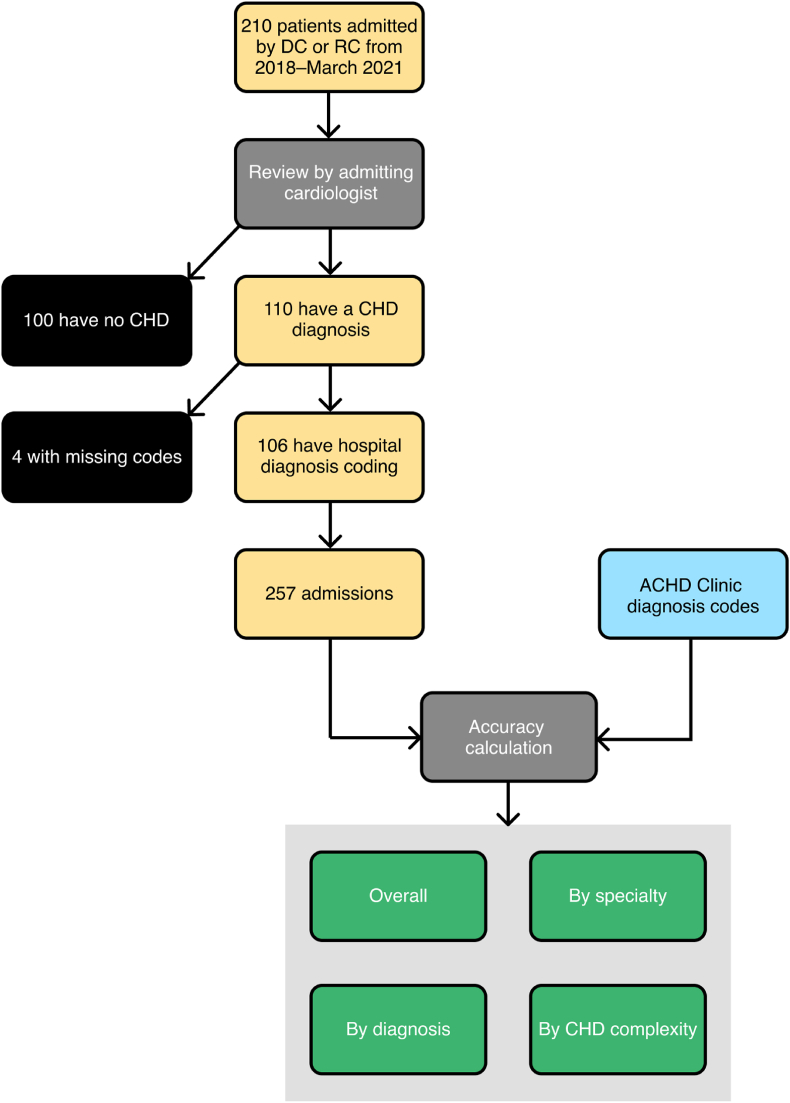
Patient flow and analysis pathway.

From all hospital inpatient admissions at the Royal Prince Alfred Hospital in Sydney, Australia, we selected those patients admitted under one of the specialist adult CHD cardiologists (DC or RC) from January 2018 to March 2021 (n ​= ​210; see Fig. 1). These patients underwent a review by the admitting cardiologist to determine whether they were CHD patients. Of the initial selection, 110 patients had CHD (the others had non-CHD related diagnoses) and these were selected for further investigation. Of these, 106 had discharge coding summaries available to be exported. These 106 patients had a combined 257 admissions over the study period with an average of 2.4 admissions *per* patient. The discharge coding summaries for each admission were exported for use in the following analysis. The ICD-10-AM discharge diagnosis codes were manually compared against the patients’ true diagnoses as entered in the dedicated Adult CHD (ACHD) Research database by specifically trained CHD coding specialists and manually verified by the team of ACHD cardiologists. The ACHD Research database stores diagnosis codes using the European Paediatric Cardiac Code matched to ICD-10-AM.

### 2.2 Analysis

For each patient admission, three pre-specified accuracy metrics were analyzed. First, we checked whether there was any CHD diagnosis present in the discharge coding summary associated with that admission. Next, we checked whether there were *any* CHD codes that matched with the diagnostic codes in the ACHD research database. Finally, we checked whether *all* of the codes in the ACHD database were present in the discharge coding summary. The proportion of admissions fulfilling these criteria constituted our definition for coding accuracy. Proportions were calculated for the cohort as a whole; separately for patients stratified into simple, moderate and complex CHD according to the Bethesda criteria [8]; and separately depending on whether the patients were admitted under a cardiovascular specialty (Cardiology, Vascular Surgery or Cardiothoracic Surgery), or under a non-cardiovascular specialty. Chi-squared tests were performed for cardiovascular versus non-cardiovascular specialties within each accuracy metric. Sensitivity, specificity and positive predictive value were also calculated for each diagnosis code present in the discharge coding summaries of at least five patients.

While the accuracy of discharge codes *per admission* was the focus of this analysis, we also calculated the same three accuracy metrics *per patient*, with all admissions for that patient within the study period combined.

## 3 Results

**Fig. 2:**
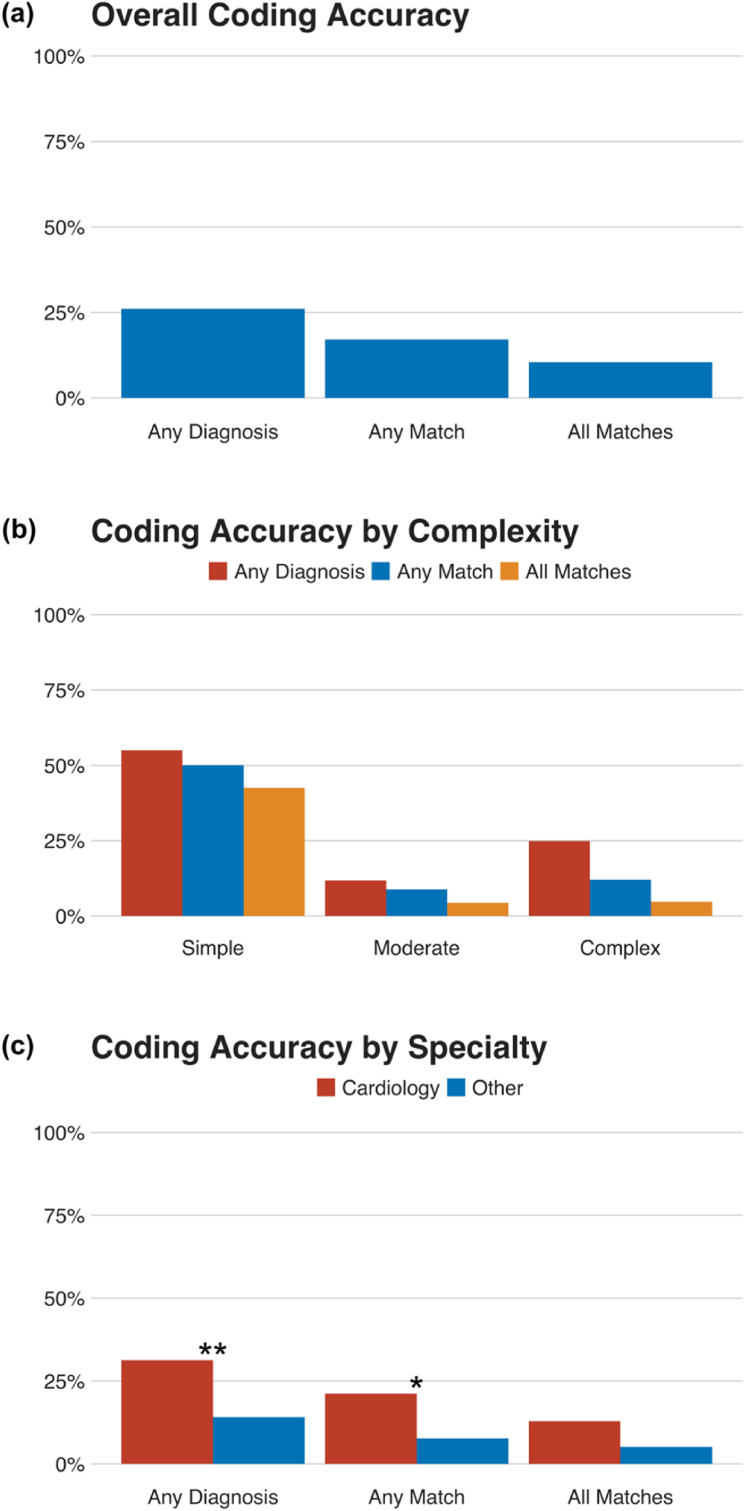
(a) Informal performance metrics for discharge coding summaries. (b) Informal performance metrics for discharge coding summaries, separated by lesion complexity according to the Bethesda criteria [8]. (c) Informal performance metrics for discharge coding summaries, separated by admitting specialty. For each performance metric, cardiovascular specialties are compared to non-cardiovascular specialties using Chi-squared tests. **∗∗**: *p* ​< ​0.01; **∗**: *p* ​< ​0.05.

Overall, 26% of discharge coding summaries contained any diagnosis of CHD, 17% contained a diagnosis matching with the “gold standard” in the ACHD clinic database, and 11% contained all the diagnoses present in the ACHD clinic database (Fig. 2a). The commonest “correct” CHD diagnoses (ICD-10 codes) were Q21.0, Ventricular septal defect (n ​= ​35); Q21.1, Atrial septal abnormality (n ​= ​31); Q20.3, Transposition of the great arteries (n ​= ​24); and Q21.3, Tetralogy of Fallot (n ​= ​16; Table 1). Note that in many cases, more than one of these correct codes were found in the same discharge coding summary.

**Table 1:** List of true diagnoses in the study cohort, as recorded in the ACHD Research database and individually verified by ACHD specialists. Patients often have multiple true diagnoses.

ICD-10 Code	Prevalence	*n*	Diagnosis
Q21.0	33.02%	35	Ventricular septal defect
Q21.1	29.25%	31	Atrial septum abnormality
Q20.3	22.64%	24	Transposition of great arteries
Q21.3	15.09%	16	Tetralogy of Fallot
Q22.1	15.09%	16	Pulmonary stenosis
Q23.9	9.43%	10	Mitral valvar abnormality
Q20.1	9.43%	10	Double outlet right ventricle
I27.8	8.49%	9	Secondary pulmonary hypertension
Q25.0	8.49%	9	Patent arterial duct
Q22.4	7.55%	8	Tricuspid atresia
Q23.1	7.55%	8	Aortic regurgitation
Q22.0	7.55%	8	Pulmonary atresia
Q20.4	6.60%	7	Double inlet atrioventricular connection
Q21.2	5.66%	6	Common atrium
Q22.5	3.77%	4	Ebstein malformation of tricuspid valve
Q26.3	2.83%	3	Partially anomalous pulmonary venous connection
Q20.8	0.94%	1	Concordant VA connections with parallel great arteries
Q25.6	0.94%	1	Supravalvar pulmonary trunk stenosis

When stratified by CHD complexity, simple patients were coded most accurately, followed by complex patients, with moderate complexity patients coded with the least overall accuracy (Fig. 2b). When separated by admitting specialty, discharge codes from admissions under cardiovascular specialties (Cardiology, Vascular Surgery or Cardiothoracic Surgery) contained at least one CHD diagnosis 31% of the time, as compared to 14% under non-cardiovascular specialties (p ​= ​0.006); a match was present in 21% of discharge coding summaries as opposed to 8% under non-cardiovascular specialties (p ​= ​0.014); finally, all matching codes were present in 13% of discharge coding summaries under cardiovascular specialties, as opposed to 5% under non-cardiovascular specialties (p ​= ​0.1; Fig. 2c).

**Fig. 3:**
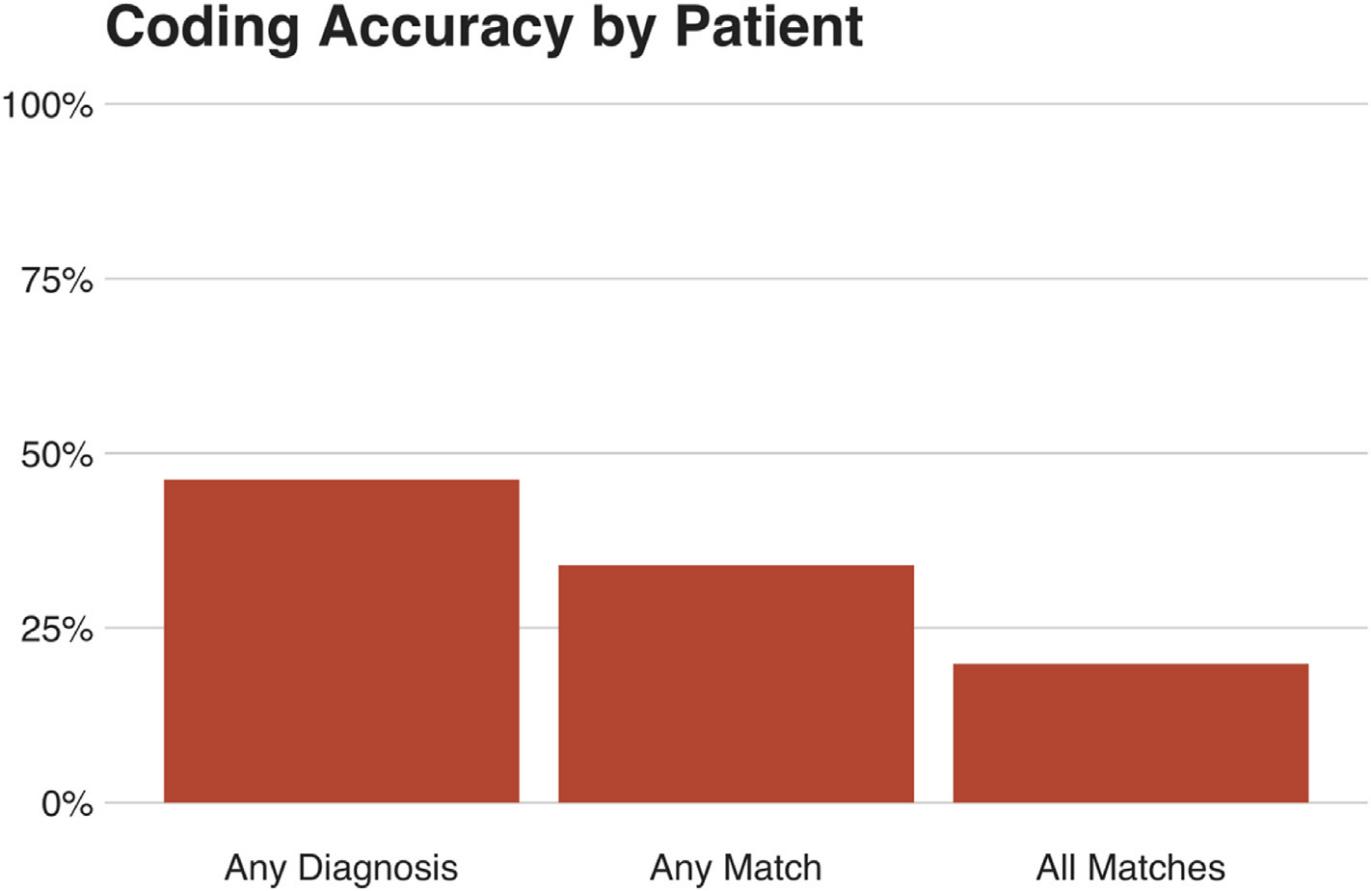
Informal performance metrics for hospital discharge coding summaries when taken as a whole, combining all admissions for each patient over the three-year study period.

When all admissions over the study period were combined for each patient, there was at least one CHD diagnosis across the study period for 46% of patients, at least one match for 34% of patients, and all appropriate diagnoses were present for 20% of patients (Fig. 3).

Sensitivity, specificity and positive predictive value for each diagnosis are presented graphically in Figure S1. Specificity for all CHD diagnoses present in at least five individuals’ discharge coding summaries was uniformly high (Mean ​= ​0.99, SD ​= ​0.01). Sensitivity, on the other hand, was variably low (Mean ​= ​0.26, SD ​= ​0.20). Positive predictive value was also highly variable due to the presence of some broad catch-all codes (e.g. “Q23.9: Congenital malformation of aortic and mitral valves, unspecified”) that were frequently used in discharge coding summaries but not in the ACHD Clinic database (Mean ​= ​0.79, SD ​= ​0.36).

## 4 Discussion

For three-quarters of admissions of patients with CHD, the associated discharge coding summary contained no CHD diagnoses at all (Fig. 2a). Even when these patients were admitted under cardiovascular specialties (Cardiology, Vascular Surgery or Cardiothoracic Surgery), two-thirds of discharge coding summaries contained no mention of CHD (Fig. 2c). Discharge coding summaries were least likely to include CHD diagnoses in cases of moderate and complex disease, with only one in five of these patients receiving any diagnosis code for CHD (Fig. 2b). Despite this, when CHD codes were contained in the summaries, they were trustworthy, with high specificity and positive predictive value (Figure S1).

When all admissions for a particular patient during the study period were combined, only 46% of patients had any CHD diagnosis recorded (Fig. 3). These results are similar to those found by Khan et al. who examined administrative data in a US setting and documented that ICD-9-based discharge coding correctly identified just 48.7% of individuals with CHD [9]. Case identification rates in cardiology departments with integrated ACHD units have been reported up to 81%, although the sensitivity for individual CHD codes remains highly variable, and generally low [10].

The surprising inadequacy of discharge coding summaries for CHD may be due to the unique difficulty of coding CHD lesions. As recently published by our group (Chami et al., 2021) the sheer variety, rarity, and complexity of CHD lesions makes accurate coding by non-specialists extremely difficult [2]. Moreover, even if cardiologists themselves were to input the discharge codes, the system used in Australian hospitals for discharge coding summaries since 1998, ICD-10-AM, often lacks the requisite detail to do so [1]. Thus, hospital discharge coders lack the tools, and may lack the expertise, to accurately document CHD lesions. This might explain, for example, ten instances of the ICD-10-AM code “Q23.9: Congenital malformation of aortic and mitral valves, unspecified” used as catch-all term for more specific diagnoses.

Given that the codes contained in these discharge coding summaries are used for healthcare planning and resource allocation, it is noteworthy that even when the discharge codes from multiple admissions over the three-year study period are combined, most CHD patients have no recorded CHD diagnosis (Fig. 3). This basic omission has potentially far-reaching consequences. Underestimating the prevalence and medical consequences of CHD means that less research and clinical funding is available for the field, hindering progress. Moreover, previous studies have found that inaccurate discharge summaries were associated with a greater prevalence of medical errors, especially on handover or re-presentation [11]. The particularly low CHD coding rates among non-cardiovascular admissions may represent a “knowledge gap” in junior doctors in such specialties. Thus, without an accurate assessment of the prevalence of CHD and importance of CHD subspecialists to manage it, health systems are unable to optimize the allocation of funding and human resources to address the problem.

It is unsurprising that lesions of moderate or great complexity were less likely to contain matching discharge codes, given the well-known difficulty of CHD coding, especially within the reductive ICD-10-AM classification system [2]. It is surprising, however, that in the majority of patients with complex CHD (and therefore great risk of morbidity and mortality regardless of presentation) no CHD diagnosis was coded at all, even over multiple presentations over three years. It appears, therefore, that CHDs are often only listed in discharge coding summaries if the primary reason for admission is an interventional treatment. Indeed, a large proportion of admissions with simple lesions were for the closure of an atrial septal defect, which was therefore coded correctly in the majority of cases. One previous study noted parenthetically that more complicated diagnoses were more likely to be diagnosed at birth, while simpler and commoner diagnoses were generally made during outpatient visits and would therefore not be found in hospital discharge coding summaries [12]. This may explain why CHDs of moderate complexity were less likely to appear in hospital discharge coding summaries than CHDs of great complexity.

Since the discharge coding summaries are based on clinical notes written during the patient’s stay, it is unsurprising that patients admitted under cardiovascular specialties with cardiovascular symptoms were more likely to have a previous CHD diagnosis featured prominently in their inpatient documentation. It nonetheless important to acknowledge and document the management of non-cardiovascular sequelae of CHD, especially in Gynecology/Obstetrics (10 admissions), Renal (8 admissions) and General Medicine (14 admissions). Failure to do so underestimates the role that CHD specialists play in the total medical care of such individuals. Regarding heart disease in general, and not specifically CHD, primary diagnoses are usually coded accurately with sensitivity and specificity between 83% and 93% (for acute myocardial infarction, arrhythmia and congestive heart failure) [13]. Comorbidities, however, have previously been found to be systematically underreported in an Australian context [14].

We believe that there are three potential reasons for the inadequacy of hospital discharge coding in the context of CHD. Firstly, the lack of specific training for CHD coding could explain the accuracy disparity between the codes in the AHCD database, which are correct and complete, and the hospital discharge summaries, which often coded CHD non-specifically if at all. More training specific to CHD coding may help improve accuracy, especially in the transition from ICD-10-AM to ICD-11. Secondly, the difference between coding accuracy in cardiovascular and non-cardiovascular admissions suggests that part of the problem may be that discharge summaries from junior doctors outside cardiology might be less clear, in terms of CHD diagnoses, making the job of the coders more challenging. More research on current pressures facing junior doctors may uncover the cause of poor-quality discharge summaries, and how to support junior doctors to improve them. Finally, we identified inadequacies in ICD-10-AM itself, which lacks sufficient detail for many lesions. This may force discharge coders to use, for example, broad terms like “Other congenital malformations of cardiac septa” to describe the particular diagnosis of “Eisenmenger syndrome”. Transitioning promptly to ICD-11, which contains more than four times as many base codes for CHD as ICD-10-AM [2], will help alleviate this issue and hopefully lead to greater detail in discharge coding summaries. Finally, we note that in certain other jurisdictions, accuracy of discharge coding could be influenced for reimbursement reasons. This is unlikely in the Australian context.

There are limitations to the current work. Patient discharge coding summaries were only available for the past three years, and so it is impossible to calculate what proportion of CHD patients have received any coded diagnosis of CHD across their lifetime, not just over the study period. Moreover, although the cohort size was relatively small, it was complete for the study’s duration and thus relatively robust and significant conclusions were able to be drawn; for example, that CHD patients admitted under cardiovascular specialties were more likely to have a diagnosis of CHD recorded in their discharge coding summary. Finally, although this work reflects a hospital with a specialty interest in CHD management, Australia’s universal health care access system suggests that these results may be generalizable across other hospitals; we acknowledge that this would require separate confirmation in future research.

In conclusion, hospital discharge coding summaries may currently be inaccurate for the estimation of CHD disease burden, as they likely systematically underestimate the prevalence of CHD. This may especially be the case when CHD patients are admitted under non-cardiovascular specialties. In order to design health policy fairly and accurately, hospital discharge coding accuracy must be considered and if feasible, improved.

## Sources of funding

No funding source to declare.

## Disclosures

Nil relevant.

## Declaration of competing interest

The authors declare that they have no known competing financial interests or personal relationships that could have appeared to influence the work reported in this paper.
