# Supplementary material for: Hospital discharge codes and substantial underreporting of congenital heart disease
Source: Int J Cardiol Congenit Heart Dis. 2022 Jan 4;7:100320. doi: 10.1016/j.ijcchd.2022.100320 (PMC11658532; doi:10.1016/j.ijcchd.2022.100320)
Supplement: Multimedia component 1 [file mmc1.docx]

**Supplemental Material**


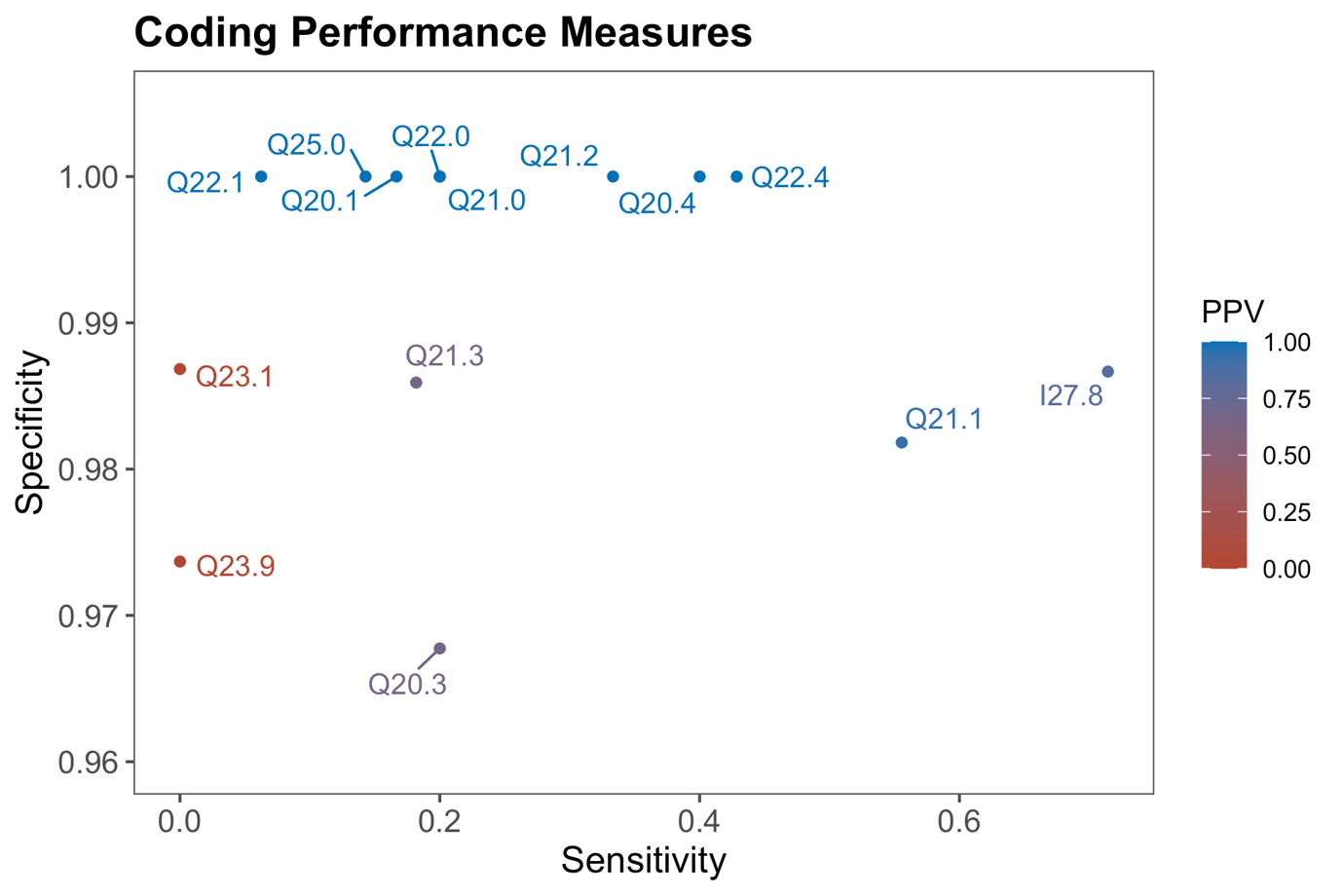


Figure S1. Sensitivity, specificity, and positive predictive value (PPV) of ICD-10 codes in hospital discharge coding summaries.
